# Hospitalist Care for Unplanned Oncology Admissions: A Mixed‐Method Analysis of Oncology and General Hospitalist Outcomes and Processes

**DOI:** 10.1002/cam4.71679

**Published:** 2026-02-24

**Authors:** Megan A. Mullins, Sanah Ladhani, Abril Carrillo, Emily C. Repasky, Michael Wu, Bella Etingen, Emre Tarhan, Navid Sadeghi, Jason B. Fleming, Suzanne D. Conzen, Arthur S. Hong

**Affiliations:** ^1^ Department of Health Economics, Systems, and Policy, Peter O'Donnell Jr. School of Public Health University of Texas Southwestern Medical Center Dallas Texas USA; ^2^ Department of Internal Medicine University of Texas Southwestern Medical Center Dallas Texas USA; ^3^ Harold C. Simmons Comprehensive Cancer Center UT Southwestern Medical Center Dallas Texas USA; ^4^ Division of Hematology and Oncology University of Texas Southwestern Medical Center Dallas Texas USA; ^5^ Peter O'Donnell Jr. School of Public Health University of Texas Southwestern Medical Center Dallas Texas USA; ^6^ Research and Development Service Dallas VA Medical Center Dallas Texas USA; ^7^ Parkland Health Dallas Texas USA; ^8^ Department of Surgical Oncology University of Texas Southwestern Medical Center Dallas Texas USA

**Keywords:** mixed‐methods, oncology hospitalist, outpatient, team communication, teaming

## Abstract

**Purpose:**

To compare patient outcomes across oncology hospitalist (OH) and general hospitalist (GH) teams and understand related team practices.

**Methods:**

Using an explanatory sequential mixed‐methods design, we compared unplanned inpatient admissions to the GH or OH teams at our academic medical center between 2018 and 2022 using propensity‐score matching to balance characteristics across teams. Primary outcomes included outpatient oncology follow‐up, 30‐day readmission, and discharge to hospice. Outcomes were modeled with multivariable logistic regression, adjusted for age, race, and comorbidity. We conducted semi‐structured interviews with OH (*n* = 6), GH (*n* = 7), and oncology consult team (*n* = 4) clinicians. Themes were identified through rapid qualitative analysis and consensus discussions between two qualitative researchers.

**Results:**

The matched sample included 1082 GH and 361 OH team admissions. Median length of stay was 5 days (IQR 3,8) for GH and 6 days for OH (IQR 4, 10) (*p* < 0.01). The OH team had higher adjusted odds of discharge to hospice (aOR: 1.9, 95% CI: 1.1–3.5), outpatient oncology follow‐up within 30 days (aOR: 4.2, 95% CI: 3.1–5.6), but also 30‐day readmissions (aOR: 1.8, 95% CI: 1.3–2.5) compared to the GH team. The OH team communicated directly with outpatient oncology throughout hospitalizations and sent discharge summaries with follow‐up recommendations. In contrast, the GH team relied on the consulting oncology team to communicate with outpatient oncologists and had variable follow‐up practices.

**Conclusion:**

Engagement with outpatient oncologists throughout patient admissions may contribute to more effective discharge planning. Inpatient teams can use this strategy to help improve hospital transitions of care for patients with cancer.

## 1 Introduction

Patients with cancer experience high rates of unplanned hospitalization, particularly near the time of cancer diagnosis and the end of life [1, 2, 3, 4, 5, 6, 7, 8]. These admissions drive cost variability, and most commonly occur due to infections and treatment complications [1, 2, 3, 6, 7, 8, 9]. Given the increasing complexity of cancer treatment and longer expected survival for patients with advanced cancer, hospital visit volumes will continue to grow [6, 10, 11]. However, many oncologists report that inpatient care is not the best use of their clinical time [12]. Such perceptions, coupled with the growing oncology workforce shortage, spurred increasing interest in hospitalist‐led management of inpatient cancer care [13, 14].

Hospitalist management of cancer‐related admissions has gained favor in recent years due to the value, cost‐effectiveness, and quality of care that hospitalists provide [15]. Although a growing number of academic institutions have developed oncology hospitalist programs, the structures of such programs vary, ranging from hospitalist‐oncologist co‐management to dedicated oncology hospitalists (OH) [15, 16, 17]. On traditional oncologist‐led services, an inpatient oncologist serves as the attending‐of‐record, whereas hospitalist‐led models have a hospitalist attending‐of‐record and oncology input is provided through consultation or communication with the patient's outpatient oncology team. Studies have compared hospitalist teams to oncologist‐led inpatient teams, but research directly comparing hospitalists who exclusively care for oncology patients to general hospitalists (GH) who manage a broader inpatient population is lacking [15, 16, 17]. In addition, effective team processes and communication are recognized as essential to high‐quality cancer care; however, they have not been evaluated in the OH model [18].

To address these gaps, we conducted a mixed‐method study comparing GH and OH teams at a North Texas Comprehensive Cancer Center (CCC). Our goal was to evaluate differences in clinical outcomes and explore how team structure and practices may influence care delivery for hospitalized patients with cancer.

## 2 Methods

### 2.1 Design and Setting

We used an explanatory sequential mixed‐method approach to understand differences in clinical outcomes for OH and GH teams at a tertiary care hospital affiliated with a North Texas CCC. OH teams are general internal medicine physicians in the Division of Oncology, whereas GH teams are general internal medicine physicians in Hospital Medicine. OH teams primarily care for CCC patients. All other patients with cancer go to the GH service for unplanned admissions. When OH teams reach their cap of patients, any additional CCC patients are placed on GH teams. Quantitative analytic findings informed qualitative interview guide development, with the goal of understanding team characteristics and practices that may contribute to outcome differences. The study was approved by the UT Southwestern (UTSW) Institutional Review Board (IRB #STU‐2023‐0085 and IRB #STU‐2023‐1064).

### 2.2 Data Sources

We analyzed data from the CCC cancer registry (November 2015–December 2022) and electronic health record (EHR) (November 2018–December 2022). Semi‐structured interviews lasting 30–60 min were conducted from April to June 2025 via videoconferencing by two qualitatively‐trained researchers (ER, MM). Interviews were video recorded and transcribed verbatim. All participants gave verbal consent to be interviewed and audio recorded.

### 2.3 Patient Cohort

We identified unplanned inpatient admissions (cancer admissions excluding planned surgery or treatment) to either the GH or OH service between November 2018 and December 2022 for patients aged ≥ 18 who had a cancer diagnosis between November 2015 and December 2022 (*n* = 13,390). We included the first unplanned admission as the index admission and excluded subsequent admissions so that each patient's first inpatient admission determined team assignment (GH vs. OH) (*n* = 8403).

### 2.4 Clinician Interviews

Because the inpatient oncology consultant team can support the GH and OH teams as they manage cancer patients on their service, eligible interviewees included individuals currently working on GH, OH, or inpatient oncology consultant teams. Recruitment was facilitated by GH and OH leaders via introductory emails. Prospective participants were then contacted by the study team via email. Interested participants were scheduled for interviews. All participants were assured that the interviews were confidential and their responses would be deidentified. A total of nineteen clinicians completed an interview. Two also completed a subsequent follow‐up interview for clarification purposes, and two did not respond after three contact attempts to complete a subsequent follow‐up interview.

### 2.5 Measurements

#### 2.5.1 Quantitative Outcomes

Primary outcomes included 30‐day readmission, discharge to hospice during index admission, and outpatient oncology follow‐up within 30 days of discharge. Readmission was defined as an emergency department visit, observation stay, or inpatient admission in the 30 days post discharge. Secondary outcomes included the proportion of patients who received an oncology or palliative care consult during their admission (at all and within 24 h of hospitalization, identified by completion of a consult note) and length of stay (LOS).

#### 2.5.2 Quantitative Exposure

At the time of admission, patients with cancer were assigned to either an OH (*N* = 361) or GH (*N* = 8042) team based first on whether they were a CCC patient then on patient census. During the study period, the OH service consisted of two teams staffed by six different hospitalist physicians (2 on service at a time), caring exclusively for patients with cancer. The GH service included 30 hospitalists (15 on service at a time) who cared for patients with and without cancer.

#### 2.5.3 Quantitative Covariates

Patient clinical and sociodemographic characteristics were ascertained from the cancer registry and the EHR at the time of index admission. Covariates included age (categorized as 18–39, 40–64, and ≥ 65 years), sex (Female/Male), race (White, Black, Other), comorbid conditions, re‐admission risk (tertile of Elixhauser Comorbidity Index Risk of 30‐day readmission score), cancer type (breast, lung, prostate, colorectal, and other cancer), and year of admission [19].

#### 2.5.4 Qualitative Data Collection

The interview guide was developed and piloted with a GH team member with OH experience (SL). The guide included questions about roles, between‐team interactions, discharge processes, and care coordination.

### 2.6 Statistical Analysis

To balance disease severity and treatment complexity across patients seen by OH and GH teams, patients were propensity score matched on age, sex, race, comorbid conditions, and metastatic cancer diagnosis (Table S1). A greedy nearest‐neighbor matching approximation with a maximum allowable distance caliper of 0.2 was used.

To examine the association between hospitalist team type and outcomes, we first assessed differences in bivariate distributions using chi‐square analyses for (categorical measures) and Mann–Whitney U tests for (continuous measures) due to skewed distributions. We then used multivariable‐adjusted logistic regression to estimate the association between hospitalist team type and outcomes (30‐day readmissions, discharges to hospice, follow‐up with outpatient oncology), adjusting for age, patient race, re‐admission risk, year of admission, and consultations (oncology, palliative care). Analyses were conducted in SAS version 9.4 and a two‐tailed *p* value < 0.05 was considered statistically significant.

### 2.7 Qualitative Analysis

Interviews were analyzed as they were completed, using rapid qualitative analysis to facilitate timely and systematic synthesis of findings [20, 21]. Immediately following each interview, data were summarized and organized by role in structured rapid analysis matrices informed by the interview guide domains, facilitating identification of nascent patterns [22]. Two qualitatively trained researchers (ER, MM) conducted interviews. Both researchers listened to interview recordings; ER populated the analysis matrix, MM reviewed, and the two met to compare interpretations, identify discrepancies or missing information, and resolve differences through discussion until consensus was reached [23, 24]. Using the rapid analysis matrix, findings were triangulated across clinician roles and integrated with quantitative findings. We maintained an audit trail of matrix iterations and analytic decisions generated during these meetings to support dependability and confirmability [25, 26]. We assessed thematic saturation by tracking the emergence of new themes across successive interviews [27, 28]. In lieu of formal member checking, we sent follow‐up questions to participants via email for clarification as needed. The study is reported according to the COnsolidated criteria for REporting Qualitative research (COREQ) checklist [29].

## 3 Results

Standardized mean differences were used to assess post‐matching balance across covariates, with an absolute difference less than or equal to 0.01 for all matching variables (Table S1). The final matched sample included 1443 CCC patients who had an unplanned inpatient admission (1082 GH, 361 OH). Most patients were over the age of 65 (52.9%), male (52.7%), and white (73.0%) (Table 1).

**TABLE 1:** Clinical and sociodemographic characteristics of a matched cohort of North Texas Comprehensive Cancer Center Patients who had an unplanned inpatient admission between 2018 and 2022.

	General hospitalist patients (*n* = 1082)	Oncology hospitalist patients (*n* = 361)	Total (*n* = 1443 (%))
Age			
Age 18–39	50 (4.6)	18 (5.0)	68 (4.7)
Age 40–64	459 (42.4)	153 (42.4)	612 (42.4)
Age 65+	573 (53.0)	190 (52.6)	763 (52.9)
Sex			
Female	512 (47.3)	171 (47.4)	683 (47.3)
Male	570 (52.7)	190 (52.6)	760 (52.7)
Race			
White	790 (73.0)	263 (72.9)	1053 (73.0)
Black	188 (17.4)	62 (17.2)	250 (17.3)
Other race	104 (9.6)	36 (10.0)	140 (9.7)
Cancer type			
Breast cancer	56 (5.2)	19 (5.3)	75 (5.2)
Lung cancer	103 (9.5)	28 (7.8)	131 (9.1)
Prostate cancer	89 (8.2)	9 (2.5)	98 (6.8)
Colon cancer	25 (2.3)	13 (3.6)	38 (2.6)
Other cancer	809 (74.8)	292 (80.9)	1101 (76.3)
Elixhauser risk of readmission index			
Tertile 1	628 (58.0)	206 (57.1)	834 (57.8)
Tertile 2	47 (4.3)	23 (6.4)	70 (4.8)
Tertile 3	407 (37.6)	132 (36.6)	539 (37.4)
Year			
2018	76 (7.0)	1 (0.3)	77 (14.6)
2019	397 (35.7)	23 (6.4)	420 (29.1)
2020	260 (24.0)	89 (24.7)	349 (24.2)
2021	238 (22.0)	148 (41.0)	386 (26.7)
2022	111 (10.3)	100 (27.7)	211 (14.6)

The median LOS was 6 days (range 4,10) for patients seen by OH teams and 5 days for patients seen by GH teams (range 3,8) (*p* < 0.01) (Table 2). OH teams discharged more patients to hospice than GH teams (10.5% vs. 3.2%, *p* ≤ 0.01). More OH team patients received an oncology consultation (17.2%, *n* = 62 vs. 14.1%, *n* = 153, *p* < 0.01) and/or a palliative care consult (23.0%, *n* = 83 vs. 7.6%, *n* = 82, *p* = 0.03) during their admission compared to GH team patients.

**TABLE 2:** Admission characteristics for oncology and general hospitalist teams in a matched cohort of North Texas comprehensive cancer center patients who had an unplanned admission 2018–2022.

Categorical variables	General hospitalist admissions (*n* = 1082)	Oncology hospitalist admissions (*n* = 361)	*p* value
	*N* (%)	*N* (%)	
Oncology consults during admission			
Oncology consult ordered	207 (19.1)	115 (31.9)	< 0.01
Oncology consult completed	153 (14.1)	62 (17.2)	< 0.01
Oncology consult order timing			
Ordered in first 24 h of admission	57 (5.3)	53 (14.7)	0.70
Ordered after first 24 h of admission	150 (13.9)	62 (17.2)	< 0.01
Palliative care consults during admission			
Palliative care consult ordered	103 (9.5)	126 (34.9)	0.13
Palliative care consult completed	82 (7.6)	83 (23.0)	0.03
Palliative care consult order timing			
Ordered in first 24 h of admission	11 (1.0)	15 (4.2)	0.43
Ordered after first 24 h of admission	92 (8.5)	111 (30.7)	0.18
Discharge disposition			
Death	11 (1.0)	8 (2.2)	< 0.01
Hospice	35 (3.2)	38 (10.5)	
Home	928 (85.8)	286 (79.2)	
Other	108 (10.0)	29 (8.0)	

Continuous variables	Median (range)	Median (range)	*p* value
Overall length of stay	5 (3.0, 8.0)	6 (4.0, 10.0)	< 0.01
Length of stay by discharge disposition			
Days to death	18 (5.0, 26.0)	17.5 (8.5, 22.0)	0.90
Days to home	3 (2.0, 6.0)	4.5 (3.0, 7.0)	< 0.01
Days to other	10 (6.0, 18.0)	11 (7.0, 20.0)	0.37
Days to hospice	7 (4.0, 15.0)	9.5 (5.0, 16.0)	0.31
Time to consult			
Days to oncology consult order	2 (1.0, 3.0)	2 (1.0, 2.0)	< 0.01
Days to oncology consult completion	2 (1.0, 3.0)	2 (1.0, 3.0)	0.35
Days to palliative care consult order	3 (2.0, 7.0)	2 (2.0, 5.0)	0.01
Days to palliative care consult completion	3 (2.0, 6.0)	2 (2.0, 5.0)	0.07

**FIGURE 1:**
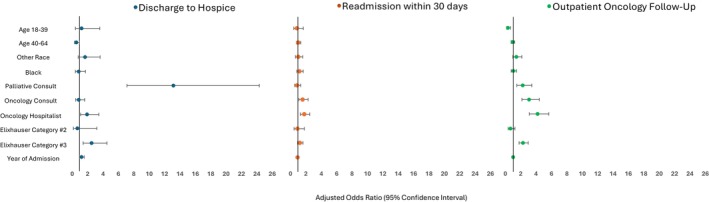
Adjusted odds ratios and 95% confidence intervals of the association between hospitalist team type and discharge quality outcomes in a matched cohort of a North Texas Comprehensive Cancer Center Patients who had an unplanned admission 2018–2022. Models are adjusted for age, patient race, palliative and oncology consultation, year of admission, and patient Exlixahuser readmission risk tertile. Reference groups are age 65+, White, No palliative care consult, No oncology consult, general hospitalist, year and Elixhauser Index Readmission Risk Tertile 1.

The OH (vs. GH) team had fewer readmissions in the 1–7 days post discharge (2.8% vs. 5.3%, *p* ≤ 0.01), but more readmissions in the 8–30 days post‐discharge (25.8% vs. 16.7%, *p* = 0.04) (Table 3). OH (vs. GH) team patients also had more outpatient oncology follow‐up in the month after discharge (62.9% vs. 29%, *p* < 0.01). In adjusted models, OH team patients were almost twice as likely to be discharged to hospice than GH team patients (Odds Ratio (OR) 1.9, Confidence Interval (CI) 1.1, 3.5). OH (vs. GH) team patients had 60% higher odds of 30‐day readmission (OR 1.8 CI 1.3, 2.5) and were four times more likely to have an outpatient oncology visit following their discharge (OR 4.2, CI 3.1, 5.6) (Figure 1).

**TABLE 3:** Follow‐up characteristics for oncology hospitalist and general hospitalist team admissions in a matched cohort of North Texas comprehensive cancer center patients who had an unplanned admission 2018–2022.

	General hospitalist admissions (*n* = 1082), *N* (%)	Oncology hospitalist admissions (*n* = 361), *N* (%)	*p* value
Re‐admissions			
1–7 days post‐discharge	57 (5.3)	10 (2.8)	< 0.01
8–30 days post discharge	181 (16.7)	93 (25.8)	0.04
Outpatient oncology follow‐up visit			
Within 11–30 days	132 (12.2)	76 (21.1)	< 0.01
Within 1–10 days	186 (17.2)	151 (41.8)	< 0.01
Overall for 30 days post discharge	318 (29.4)	227 (62.9)	< 0.01

Days to outpatient oncology follow‐up visit	Median (range)	Median (range)	*p* value
Within 1–10 days	5 (2.0, 8.0)	3 (1.0, 6.0)	< 0.01
Within 11–30 days	15.5 (13.0, 20.5)	16 (13.0, 21.0)	0.478
Overall	9 (4.0, 14.0)	6 (2.0, 13.0)	< 0.001

To better understand quantitative findings, we completed semi‐structured interviews with 17 clinicians including GH (*n* = 7), OH (*n* = 6), and inpatient oncology consult (*n* = 4) team members. Four themes were identified regarding the care and coordination of unplanned cancer admissions: (1) patient acuity and team coordination, (2) challenges consulting oncology, (3) discharge and follow‐up appointments, and (4) hospice discharges.

### 3.1 Team Coordination and Patient Acuity

Both GH and OH team members described patients with cancer as having greater complexity and requiring more cross‐team coordination than other patients.

I think cancer patients require more multidisciplinary… groups to join together to help them… And then on top of that many times they have a lot of pain… their pain is more complicated… I usually have to involve the palliative care team as well. And so it's just more complicated in that there's different teams that need to be involved and it's harder to kind of all coordinate their care GH0429

Our patients are a little bit more complicated when it comes to acute and chronic problems, social work…coordination. We often involve a palliative care team… other ones tend to be patients who are severely malnourished, weak. We use the term “failure to thrive,” not safe to go home… OH0507

### 3.2 Challenges Consulting Oncology

While OH team members describe themselves as “a bridge between the primary oncologist and the patient,” communicating directly with outpatient clinicians (primarily by email), GH team members reported relying on the oncology consult team as the “middleman” between GH and outpatient oncology (consult orders in Table 2). Though some GH team members reported no issues consulting oncology, others encountered challenges such as a lack of support.

If it is a direct question related to chemotherapy or a side effect of chemotherapy or immunotherapy, they are very helpful… If the question is more regarding disease progression and goals of care discussion, which happens I would say most of the time, more than 50% of the oncology patients that get admitted, I think we don't get as much support from the oncology team. I don't blame them, it's because usually it's not [the patient's] primary oncologist on the consult team, so they are themselves communicating with the primary oncologist who hasn't seen the patient… those patients then experience a longer length of stay. GH0513

A lot of times when I call them, I don't always get a warm reception… it's usually the PAs on call and they kinda look at the chart and usually they try to deflect the consult if it's not something complicated. So it's not really encouraging… they don't really encourage you to consult. GH0506

A GH team member and inpatient oncology consult member described delays in the team's responsiveness due to the consult attending oncologist being highly specialized in only certain cancers.

Cumbersome… the attendings that are covering the consult service… 98% of them are like sub‐specialized… if they haven't treated colon cancer in 12+ years, and they've only treated breast, and we get a new diagnosis… we do have to reach out to the GI clinic and get their input, make sure we're not missing anything… we end up having to reach out to their primary oncologist who may or may not be able to quickly respond, so that could be a little bit of a roadblock into getting quick responses. OAP0601

### 3.3 Discharge and Follow‐Up Appointments

OH team members and inpatient oncology consultants differed from GH team members in their discharge communication and follow‐up coordination as well. Inpatient oncology consultants reported consistently sending discharge emails or summaries to outpatient oncologists detailing follow‐up needs including chemotherapy schedule changes, scans, and lab work. Most OH team members also notified outpatient oncologists of patient discharge or forwarded discharge summaries, though only some specified follow‐up recommendations.

…the APPs [advance practice providers], we send discharge emails on the people who are not on the hospital oncology [OH] teams. We route the discharge summary, we look to see when their next follow‐up is, and if it's too far out, we'll put in that e‐mail… and then we put in our recommendations for follow‐up. OAP05281

…whenever they're ready to be discharged, we give them a hard stop that, “OK, this person is being discharged.” And like if their appointment is upcoming or… chemo's upcoming, then we kind of give them heads up that this is the current schedule, because this person was here for such and such… you might want to reschedule or repeat blood work or… want to change your plans. OH0505

In contrast, GH team members had mixed responses regarding discharge communication. Many noted the patient navigator, care coordinator, or oncology consult team were responsible for scheduling follow‐ups. One GH team member noted being unsure whether outpatient oncology “wants to be kept up to date” while their patients are admitted of if that might “impede their workflow.”

### 3.4 Hospice Discharge

Participants across team types described hospice discharge as a standard process, routinely consulting care coordinators or social workers to facilitate hospice transitions once the patient and family decided upon hospice care. However, two consult inpatient oncology consultants noted that waiting for confirmation from outpatient oncologists that “this is a hospice‐appropriate patient” could delay hospital discharge.

## 4 Discussion

Patients who received care from the OH team members were more likely to have outpatient oncology follow‐up and discharge to hospice compared to patients who received care from GH team members. Our qualitative findings suggested that differences in follow‐up and hospice discharge may be due to OH teams having direct engagement with outpatient oncologists throughout admissions and upon discharge (via discharge summaries and follow‐up planning). Interviews across team types suggested that increasing treatment complexity required more team coordination as well. To our knowledge, this is the first study to assess team interactions in this growing model of oncology care.

Prior studies have found similar LOS, readmissions, infections, DNR orders, and discharge disposition when comparing OH teams to oncologist‐led inpatient teams; however, this is the first study to compare OH teams to GH teams [15, 16, 17]. In our study, GH teams had shorter median LOS (1 day) compared to OH teams. This difference may reflect higher rates of consultation and hospice discharge among OH patients, which take more time. Although we saw slightly higher odds of readmission for OH compared to GH team patients in adjusted models, OH teams had a lower readmission rate in the week post discharge. These findings may reflect more preventable readmissions among GH patients, but more work is needed to disentangle what is driving these differences [30].

Similar to prior work, OH teams discharged around 10% of patients to hospice, which was significantly more than GH teams [31]. Interviews suggested a standard hospice discharge process across both teams. However, deciding a patient is hospice appropriate might require coordinating across several teams and delay discharge overall. One GH team member noted a need for more oncology support with goals of care conversations and disease progression, which may have contributed to fewer GH hospice discharges.

Despite the oncology consult team connecting GH teams to outpatient oncology, GH team patients had fewer oncology consults than OH team patients. Both inpatient oncology consultants and GH team members described challenges related to coordinating with outpatient oncology. In contrast, OH team members described more consistent communication with outpatient oncology, culminating in an actionable discharge summary to the outpatient setting. Although several interviewees noted that all GH team members communicating directly with outpatient oncology could result in “too many cooks in the kitchen,” challenges also arose when consults required outpatient oncology input. Exploratory qualitative findings suggest direct GH‐outpatient oncology communication may be warranted to improve efficiency and coordination in specific cases such as hospice.

A strength of this study is the mixed‐method design that provides insights on team‐based contributors to the quantitative outcomes we identified. Although we were able to interview nearly all the OH and many GH and oncology consult team members, we did not have the perspectives of outpatient oncology clinicians or consult team attending oncologists. Although we identified several important themes related to coordination across teams, we will need to speak to these key groups prior to implementing solutions to address identified gaps. A second limitation is that matching was limited to reliable electronic health record data elements, so we were unable to account for patient socioeconomic status or unmeasured confounders of team assignment, which could bias findings. However, the matching factors used (race, sex, age, cancer type, comorbidities, metastatic cancer), and the re‐admission risk we adjusted for are highly correlated with patient socioeconomic status. Given how patients are assigned to teams and matching to balance any remaining complexity differences across teams, we expect remaining bias to be minimal and non‐differential. Third, differences in readmission and length of stay can reflect structural differences, including differences in discharge timing, closer outpatient follow‐up, or patient complexity, which are outside of clinical team control; though we used rigorous analytic strategies to balance this where possible. Fourth, we could not quantify revisits that were ED/obs versus re‐admissions, so our re‐admission rates are more inclusive than CMS definitions. We also could not quantify delays in ascertaining hospice appropriateness across teams given available data. Future studies disentangling types of re‐visits and delays throughout the hospice enrollment process are needed. Finally, this is a single‐institution CCC study, which limits generalizability. Although community hospitals likely do not have OH teams, GH or oncology services can also employ communication and care coordination tactics highlighted in our study.

## 5 Conclusion

As cancer care increases in complexity, thoughtful coordination across teams is required. Both the OH and GH team models require input from outpatient oncology, and more work is needed to optimize oncology consult service in the era of highly specialized oncology treatment.

## Author Contributions

**Megan A. Mullins:** conceptualization (equal), data curation (equal), funding acquisition (equal), investigation (equal), methodology (equal), project administration (equal), writing – original draft (equal), writing – review and editing (equal). **Sanah Ladhani:** conceptualization (equal), writing – review and editing (equal). **Abril Carrillo:** writing – original draft (equal), writing – review and editing (equal). **Emily C. Repasky:** data curation (equal), formal analysis (equal), writing – review and editing (equal). **Michael Wu:** data curation (equal), formal analysis (equal), writing – review and editing (equal). **Bella Etingen:** validation (equal), writing – review and editing (equal). **Emre Tarhan:** conceptualization (equal), writing – review and editing (equal). **Jason B. Fleming:** conceptualization (equal), writing – review and editing (equal). **Navid Sadeghi:** validation (equal), writing – review and editing (equal). **Suzanne D. Conzen:** conceptualization (equal), writing – review and editing (equal). **Arthur S. Hong:** conceptualization (equal), formal analysis (equal), investigation (equal), methodology (equal), project administration (equal), writing – review and editing (equal).

## Funding

Research reported in this publication was supported by the National Center for Advancing Translational Sciences of the National Institutes of Health under award Number UL1 TR003163. The content is solely the responsibility of the authors and does not necessarily represent the official views of the NIH (National Institutes of Health).

## Conflicts of Interest

The authors declare no conflicts of interest.

## Supporting information


**Table S1:** Post‐matching standardized mean differences for patient characteristics in a matched cohort of Simmons Comprehensive Cancer Center Patients who had an unplanned admission 2018–2022.

## Data Availability

The data cannot be made publicly available because they contain information that could compromise the privacy of research participants.

## References

[1] J. F. Rico , J. M. Caterino , J. A. Stephens , et al., “Variables Associated With Admission Rates Among Cancer Patients Presenting to Emergency Departments: A CONCERN Group Study,” Emergency Cancer Care 2, no. 1 (2023): 7, 10.1186/s44201-023-00022-z.

[2] J. M. Caterino , D. Adler , D. D. Durham , et al., “Analysis of Diagnoses, Symptoms, Medications, and Admissions Among Patients With Cancer Presenting to Emergency Departments,” JAMA Network Open 2, no. 3 (2019): e190979, 10.1001/jamanetworkopen.2019.0979. 30901049 PMC6583275

[3] R. L. Whitney , J. F. Bell , D. J. Tancredi , et al., “Unplanned Hospitalization Among Individuals With Cancer in the Year After Diagnosis,” Journal of Oncology Practice/ American Society of Clinical Oncology 15, no. 1 (2019): e20–e29, 10.1200/JOP.18.00254. PMC7010432 30523749

[4] Z. R. Moore , N. L. Pham , J. L. Shah , et al., “Risk of Unplanned Hospital Encounters in Patients Treated With Radiotherapy for Head and Neck Squamous Cell Carcinoma,” Journal of Pain and Symptom Management 57, no. 4 (2019): 738–745.e3, 10.1016/j.jpainsymman.2018.12.337. 30610892

[5] M. A. Mullins , J. J. Ruterbusch , P. Clarke , S. Uppal , L. P. Wallner , and M. L. Cote , “Trends and Racial Disparities in Aggressive End‐Of‐Life Care for a National Sample of Women With Ovarian Cancer,” Cancer 127, no. 13 (2021): 2229–2237, 10.1002/cncr.33488. 33631053 PMC8195844

[6] M. Rubens , S. Appunni , A. Saxena , et al., “Trend and Burden of Adult Cancer‐Related Hospitalizations in the United States,” Scientific Reports 15, no. 1 (2025): 13388, 10.1038/s41598-025-97310-x. 40251325 PMC12008368

[7] K. R. Yabroff , E. B. Lamont , A. Mariotto , et al., “Cost of Care for Elderly Cancer Patients in the United States,” Journal of the National Cancer Institute 100, no. 9 (2008): 630–641, 10.1093/jnci/djn103. 18445825

[8] J. L. Warren , K. R. Yabroff , A. Meekins , M. Topor , E. B. Lamont , and M. L. Brown , “Evaluation of Trends in the Cost of Initial Cancer Treatment,” Journal of the National Cancer Institute 100, no. 12 (2008): 888–897, 10.1093/jnci/djn175. 18544740 PMC3298963

[9] V. W. Loerzel , R. B. Hines , C. W. Deatrick , P. I. Geddie , and J. M. Clochesy , “Unplanned Emergency Department Visits and Hospital Admissions of Older Adults Under Treatment for Cancer in the Ambulatory/Community Setting,” Supportive Care in Cancer 29, no. 12 (2021): 7525–7533, 10.1007/s00520-021-06338-y. 34105026

[10] J. L. Caswell‐Jin , S. K. Plevritis , L. Tian , et al., “Change in Survival in Metastatic Breast Cancer With Treatment Advances: Meta‐Analysis and Systematic Review,” JNCI Cancer Spectrum 2, no. 4 (2018): pky062, 10.1093/jncics/pky062. 30627694 PMC6305243

[11] E. J. Lehrer , K. C. Stoltzfus , B. M. Jones , et al., “Trends in Diagnosis and Treatment of Metastatic Cancer in the United States,” American Journal of Clinical Oncology 44, no. 11 (2021): 572–579, 10.1097/COC.0000000000000866. 34560720

[12] K. R. Atlas , B. C. Egan , C. J. Novak , and R. Sidlow , “The Hospitalist Model and Oncology: Oncologist Opinions About Inpatient Cancer Care Delivery,” Oncologist 25, no. 12 (2020): e13465, 10.1634/theoncologist.2020-0514. 32744351 PMC8108059

[13] C. Schenkel , L. A. Levit , K. Kirkwood , et al., “State of Professional Well‐Being, Satisfaction, and Career Plans Among US Oncologists in 2023,” JCO Oncology Advances 2 (2025): e2400010, 10.1200/OA.24.00010. 39906334 PMC11789616

[14] L. N. Shulman , L. K. Sheldon , and E. J. Benz , “The Future of Cancer Care in the United States‐Overcoming Workforce Capacity Limitations,” JAMA Oncology 6, no. 3 (2020): 327–328, 10.1001/jamaoncol.2019.5358. 31971546

[15] J. G. Manzano , A. Park , H. Lin , S. Liu , and J. Halm , “Demonstrating Value: Association of Cost and Quality Outcomes With Implementation of a Value‐Driven Oncology‐Hospitalist Inpatient Collaboration for Patients With Lung Cancer,” BMJ Open Quality 8, no. 1 (2019): e000381, 10.1136/bmjoq-2018-000381. PMC6440595 30997414

[16] J. C. Morris , B. E. Gould Rothberg , E. Prsic , et al., “Outcomes on an Inpatient Oncology Service After the Introduction of Hospitalist Comanagement,” Journal of Hospital Medicine 18, no. 5 (2023): 391–397, 10.1002/jhm.13071. 36891947

[17] D. J. Koo , T. N. Goring , L. B. Saltz , et al., “Hospitalists on an Inpatient Tertiary Care Oncology Teaching Service,” Journal of Oncology Practice/ American Society of Clinical Oncology 11, no. 2 (2015): e114–e119, 10.1200/JOP.2014.000661. PMC5706125 25563702

[18] A. K. Jain , M. L. Fennell , A. B. Chagpar , H. K. Connolly , and I. M. Nembhard , “Moving Toward Improved Teamwork in Cancer Care: The Role of Psychological Safety in Team Communication,” Journal of the Pancreas: JOP 12, no. 11 (2016): 1000–1011, 10.1200/JOP.2016.013300. 27756800

[19] B. J. Moore , S. White , R. Washington , N. Coenen , and A. Elixhauser , “Identifying Increased Risk of Readmission and in‐Hospital Mortality Using Hospital Administrative Data: The AHRQ Elixhauser Comorbidity Index,” Medical Care 55, no. 7 (2017): 698–705, 10.1097/MLR.0000000000000735. 28498196

[20] A. L. Nevedal , C. M. Reardon , M. A. Opra Widerquist , et al., “Rapid Versus Traditional Qualitative Analysis Using the Consolidated Framework for Implementation Research (CFIR),” Implementation Science 16, no. 1 (2021): 67, 10.1186/s13012-021-01111-5. 34215286 PMC8252308

[21] A. B. Hamilton and E. P. Finley , “Qualitative Methods in Implementation Research: An Introduction,” Psychiatry Research 280 (2019): 112516, 10.1016/j.psychres.2019.112516. 31437661 PMC7023962

[22] J. B. Averill , “Matrix Analysis as a Complementary Analytic Strategy in Qualitative Inquiry,” Qualitative Health Research 12, no. 6 (2002): 855–866, 10.1177/104973230201200611. 12109729

[23] C. Vindrola‐Padros and G. A. Johnson , “Rapid Techniques in Qualitative Research: A Critical Review of the Literature,” Qualitative Health Research 30, no. 10 (2020): 1596–1604, 10.1177/1049732320921835. 32667277

[24] R. C. Gale , J. Wu , T. Erhardt , et al., “Comparison of Rapid vs In‐Depth Qualitative Analytic Methods From a Process Evaluation of Academic Detailing in the Veterans Health Administration,” Implementation Science 14 (2019): 11, 10.1186/s13012-019-0853-y. 30709368 PMC6359833

[25] R. S. Barbour , “Checklists for Improving Rigour in Qualitative Research: A Case of the Tail Wagging the Dog?,” BMJ 322, no. 7294 (2001): 1115–1117, 10.1136/bmj.322.7294.1115. 11337448 PMC1120242

[26] V. Braun , Successful Qualitative Research: A Practical Guide for Beginners (SAGE, 2013), accessed September 18, 2023, http://archive.org/details/successfulqualit0000brau.

[27] S. C. Weller , B. Vickers , H. R. Bernard , et al., “Open‐Ended Interview Questions and Saturation,” PLoS One 13, no. 6 (2018): e0198606, 10.1371/journal.pone.0198606. 29924873 PMC6010234

[28] G. Guest , A. Bunce , and L. Johnson , “How Many Interviews Are Enough?: An Experiment With Data Saturation and Variability,” Field Methods 18, no. 1 (2006): 59–82, 10.1177/1525822X05279903.

[29] A. Tong , P. Sainsbury , and J. Craig , “Consolidated Criteria for Reporting Qualitative Research (COREQ): A 32‐Item Checklist for Interviews and Focus Groups,” International Journal for Quality in Health Care 19, no. 6 (2007): 349–357, 10.1093/intqhc/mzm042. 17872937

[30] T. A. Gardner , L. E. Vaz , B. A. Foster , T. Wagner , and J. P. Austin , “Preventability of 7‐Day Versus 30‐Day Readmissions at an Academic Children's Hospital,” Hospital Pediatrics 10, no. 1 (2020): 52–60, 10.1542/hpeds.2019-0124. 31852723

[31] E. Prsic , J. C. Morris , K. B. Adelson , et al., “Oncology Hospitalist Impact on Hospice Utilization,” Cancer 129, no. 23 (2023): 3797–3804, 10.1002/cncr.35008. 37706601

