# Supplementary material for: Hospitalist Care for Unplanned Oncology Admissions: A Mixed‐Method Analysis of Oncology and General Hospitalist Outcomes and Processes
Source: Cancer Med. 2026 Feb 24;15(3):e71679. doi: 10.1002/cam4.71679 (PMC12932068; doi:10.1002/cam4.71679)
Supplement: Supplementary file 1 — Table S1: Post‐matching standardized mean differences for patient characteristics in a matched cohort of Simmons Comprehensive Cancer Center Patients who had an unplanned admission 2018–2022. [file CAM4-15-e71679-s001.docx]

| **Supplemental Table 1. Post-matching standardized mean differences for patient characteristics in a matched cohort of Simmons Comprehensive Cancer Center Patients who had an unplanned admission 2018-2022** | | | |
| --- | --- | --- | --- |
|  | **General (n=1082)** | **Oncology (n=361)** | **SMD** |
| **Age** |  |  |  |
| Age 18-39 | 50 (4.6) | 18 (5.0) | 0.00365 |
| Age 40-64 | 459 (42.4) | 153 (42.4) | -0.00039 |
| Age 65 + | 573 (53.0) | 190 (52.6) | -0.00326 |
| **Sex** |  |  |  |
| Male | 570 (52.7) | 190 (52.6) | -0.00049 |
| **Race** |  |  |  |
| White | 790 (73.0) | 263 (72.9) | -0.0016 |
| Black | 188 (17.4) | 62 (17.2) | -0.00201 |
| Other Race | 104 (9.6) | 36 (10.0) | 0.0036 |
| **Cancer Type** |  |  |  |
| Breast Cancer | 56 (5.2) | 19 (5.3) | -0.02404 |
| Lung Cancer | 103 (9.5) | 28 (7.8) | -0.01665 |
| Prostate Cancer | 89 (8.2) | 9 (2.5) | -0.03974 |
| Colon Cancer | 25 (2.3) | 13 (3.5) | -0.00924 |
| Other Cancer | 809 (74.8) | 292 (80.6) | 0.0906 |
| **Elixhauser Comorbidity** |  |  |  |
| CMR_AIDS | 0 (0.0) | 0 (0.0) | 0 |
| CMR_ALCOHOL | 3 (0.3) | 0 (0.0) | -0.00277 |
| CMR_AUTOIMMUNE | 1 (0.1) | 2 (0.6) | 0.00462 |
| CMR_CANCER_LYMPH | 12 (1.1) | 3 (0.8) | -0.00278 |
| CMR_CANCER_LEUK | 1 (0.1) | 0 (0.0) | -0.00092 |
| CMR_CANCER_METS | 245 (22.6) | 82 (22.7) | 0.00071 |
| CMR_CANCER_NSITU | 2 (0.2) | 0 (0.0) | -0.00185 |
| CMR_CANCER_SOLID | 154 (14.2) | 47 (13.0) | -0.01214 |
| CMR_DEMENTIA | 2 (0.2) | 0 (0.0) | -0.00185 |
| CMR_DEPRESS | 5 (0.5) | 5 (1.4) | 0.00923 |
| CMR_DIAB_UNCX | 17 (1.6) | 4 (1.1) | -0.00463 |
| CMR_DIAB_CX | 35 (3.2) | 14 (3.9) | 0.00643 |
| CMR_DRUG_ABUSE | 0 (0.0) | 0 (0.0) | 0 |
| CMR_HTN_CX | 1 (0.1) | 1 (0.3) | 0.00185 |
| CMR_HTN_UNCX | 25 (2.3) | 12 (3.3) | 0.01014 |
| CMR_LUNG_CHRONIC | 29 (2.7) | 11 (3.1) | 0.00367 |
| CMR_OBESE | 0 (0.0) | 0 (0.0) | 0 |
| CMR_PERIVASC | 21 (1.9) | 9 (2.5) | 0.00552 |
| CMR_THYROID_HYPO | 12 (1.1) | 7 (1.9) | 0.0083 |
| CMR_THYROID_OTH | 6 (0.6) | 4 (1.1) | 0.00554 |
